# Learning from Community Health Worker Programs, Big and Small

**DOI:** 10.9745/GHSP-D-20-00244

**Published:** 2020-06-30

**Authors:** Stephen Hodgins

**Affiliations:** a Editor-in-Chief, Global Health: Science and Practice Journal, and Associate Professor, School of Public Health, University of Alberta, Edmonton, Alberta, Canada.

## Abstract

Small, well-implemented, well-evaluated community health worker programs can provide useful insights and inspiration. Testing, learning, and adapting at progressively larger scale can ultimately lead to national-scale programs that achieve sustainable impact.

See related article by Nepal et al.

Currently, there is widespread enthusiasm for community health worker (CHW) programs. There is a commonly held view that these programs can make important contributions to improving population-level health outcomes, not only in areas of community-based primary health care (CBPHC)—like maternal-child health, family planning, nutrition, HIV, tuberculosis, and malaria—but also in emerging high-burden health conditions, such as hypertension and other noncommunicable diseases.^1^

But the global health community has gone through repeated waves of enthusiasm and disappointment with CHW programs in the past. The potential of such programs—demonstrated in exemplary pilot projects—has often failed to translate into impactful, institutionalized programs, operating at scale.

Learning not only from CHW programs but other systems interventions points to the inadequacy of simply developing a plan, marshaling the needed resources, and implementing the plan. To achieve sustainable impact at scale, those involved need to critically assess and learn from programs done at smaller scale and then apply those lessons, adapting their programs as they implement further.

Most of the documentation and learning published in the peer-reviewed literature on CHW programs has been based on comparatively small programs, often implementing narrow sets of interventions, over a relatively short period of time by local or international nongovernmental organizations or by university-based groups, in most instances with external funding.^2^ As a consequence of the filter of the peer-review process, much of this analytic work has been done with high levels of internal validity, but often at the cost of wider programmatic relevance (i.e., external validity). At the same time, at least some of these documented experiences have also been important inspirations for global and national-level policy makers and program developers.

A case in point is work done by Abhay and Rani Bang—in a comparatively poorly-served, rural population in eastern Maharashtra, India, consisting mostly of *aadhivasi* or “scheduled tribes”—demonstrating that well-designed and well-supported CHW programs can substantially reduce child and newborn mortality, even in challenging contexts.^3^^,^^4^ This work served as an important impetus for work worldwide, beginning about 20 years ago, seeking to drive down newborn mortality using community-based strategies (e.g., under the Saving Newborn Lives projects funded by the Bill and Melinda Gates Foundation and a series of global projects funded by the United States Agency for International Development).

In this issue of GHSP, we have a helpful new contribution by Nepal et al.,^5^ looking closely at costing of an exemplary but comparatively small-scale program in Achham, an economically disadvantaged district located in the Far-Western province in Nepal. The program introduced a new, full-time, paid cadre of CHW, working alongside the government CBPHC system. Each CHW was responsible for a catchment population of approximately 2,000. Their primary responsibility was to make home visits—targeting a variety of groups including pregnant and postpartum women and households with children aged 2 years and younger—for counseling, screening, and referral. To support them in these tasks, they were each issued smartphones equipped with mobile apps for documenting services delivered and supporting counseling and referral. In addition to conducting home visits, the CHWs also helped facilitate twice-monthly group antenatal care (ANC) meetings at the local government health post, where they worked together with project community health nurses and health post staff. Besides counseling and referral related to pregnancy, postpartum care, family planning, and childhood illness, the CHWs also provided counseling and referral for adult chronic diseases.

The article by Nepal and colleagues focused on work in 2 municipalities in Achham district (comprising a population of about 60,000), conducted by 30 CHWs who, in turn, were supported by 8 supervisory staff. This same area has been served by 21 government health posts and 1 primary health care center, with approximately 115 full-time health workers and 248 female community health volunteers. Although the project coordinated with local health services, most of the CHW functions were performed independently from the government CBPHC services, with the exception of the group ANC sessions described above. In addition, the project regularly reported its service data to the local health facilities and district and national government reporting systems. In other publications, the authors have documented evidence for significant impact of the program, including notable improvements in coverage for ANC, institutional childbirth, and postpartum family planning.^6^^,^^7^

Since this initiative began (and indeed before), Nepal has seen a progressive increase in staffing of government CBPHC services as well as wide testing and discussion of alternative models for expanding delivery of services at community and household levels; this has included a “community auxiliary nurse-midwife” model and “community health units” staffed by auxiliary nurse-midwives. As with the experience addressed in the article by Nepal et al., for both of these models, the population-to-health worker ratio has been approximately 2,000:1. The article helps to advance this discussion in Nepal and offers costing information that should prove useful for decision makers at local, provincial, and national levels as they consider funding commitments.

For those engaged in CHW work in other country settings, the article provides a good example of a costing exercise that was both methodologically robust and useful for decision makers. Furthermore, the program the authors describe has a number of features worth considering elsewhere, including continuous competency-based training and provision for close supervisory support. This article adds to a growing body of published literature, offering lessons from strong, nongovernmental organization-led CHW programs, implemented at relatively small scale but testing program design features that are potentially relevant for large government programs. Many of these lessons are reflected in the recent major World Health Organization guidance document^1^ and the CHW Assessment and Improvement Matrix (CHW-AIM).^8^

We have considerably less documentation in the peer-reviewed literature on what works *at scale* for CHW programs. As important as large government programs are, they are less easily studied, and solid documentation is sparse. A very helpful resource has recently been released on such large national CHW programs^9^; this compendium includes 29 case studies drawn from low- and middle-income countries across 3 continents. They follow a common format, helping facilitate comparisons across programs. The case studies look at a mix of CHW types, from the more professionalized end of the spectrum to less formalized community health volunteer programs.

Case studies of large-scale, institutionalized, public-sector programs—providing good contextual and systems support information and summarizing available information on their role and performance—can be particularly relevant and useful for national-level policy makers, program managers concerned about delivery of services to whole populations, and partners supporting them.

The set of studies edited by Perry is offered as an introduction to these programs; all of the cases either have authors with firsthand familiarity with the programs or are based on information and insights elicited from key informants with such experience. Certainly, there are further insights that can be gleaned from the programs documented in these case studies. In many instances, there would be value in more analytic work but that will require additional primary-level data collection. To date, relatively few national CHW programs have been comprehensively assessed. This compendium should be seen, then, as an invitation to dig deeper and learn more from these programs.

Important lessons can be learned from both smaller-scale demonstration projects and from large-scale institutionalized programs. If we are to avoid another wave of disappointment with CHW programs and to see strong CHW programs and CBPHC systems operating at national scale, we need to be looking systematically at both types of program experience: innovating and testing new ideas and approaches and looking critically at the factors determining performance as programs are implemented at large scale.
